# Supplementary material for: Modeling succinate dehydrogenase loss disorders in C. elegans through effects on hypoxia-inducible factor
Source: PLoS One. 2019 Dec 30;14(12):e0227033. doi: 10.1371/journal.pone.0227033 (PMC6936837; doi:10.1371/journal.pone.0227033)
Supplement: Attachment — Submitted filename: Response_to_Reviewers.docx [file pone.0227033.s001.docx]

**Response to Reviewers PONE-D-19-29380R1**

**Please find responses to referee comments below. Revisions have been highlighted in yellow in the marked-up copy of the revised manuscript.**

Editorial comments:

1. To comply with PLOS ONE submission guidelines, in your Methods section, please provide additional information regarding your statistical analyses.

**The following wording has been added:**

Statistical analysis

Values are expressed as mean +/- standard deviation for the indicated number of independent experiments. The statistical analysis was performed using the Student’s t-test, a one-way analysis of variance (ANOVA) test with post-hoc Tukey HSD, or a Dunnett’s test with R Studio software. A P value of less than 0.05 was considered statistically significant.

2. At this time, we ask that you please provide the source of the primers and NGM media used in this study.

**This information has been added to the materials and methods section.**

3. Thank you for including the following funding information within your acknowledgements section; "This work was supported by the Mayo Foundation, the Paradifference Foundation, and by NIH grant CA166025 (LJM). Some C. elegans strains were provided by the CGC, which is funded by the NIH Office of Research Infrastructure Programs (P40 OD010440). "

We note that you have provided funding information that is not currently declared in your Funding Statement. However, funding information should not appear in the Acknowledgments section or other areas of your manuscript. We will only publish funding information present in the Funding Statement section of the online submission form.

Please remove any funding-related text from the manuscript and let us know how you would like to update your Funding Statement. Currently, your Funding Statement reads as follows:

"The funders had no role in study design, data collection and analysis, decision to publish, or preparation of the manuscript."

The updated Funding Statement should read as follows:

This work was supported by the Mayo Foundation, the Paradifference Foundation, and by NIH grant CA166025 (LJM). Some *C. elegans* strains were provided by the CGC, which is funded by the NIH Office of Research Infrastructure Programs (P40 OD010440). The funders had no role in study design, data collection and analysis, decision to publish, or preparation of the manuscript.

4. We note that you have included the phrase “data not shown” in your manuscript. Unfortunately, this does not meet our data sharing requirements.

**This phrase has been deleted**.

Additional Editor Comments (if provided):

The present article focuses on exploring the relationship between succinate accumulation, hypoxic signaling, egg-laying behavior, and morphology in C. elegans. In the present study, authors specifically focus on egg retention in C. elegans associated with SDH-loss disorders. They hypothesize that HIF-1alpha is the main player in egg retention. Since almost entire manuscript is devoted to this problem, the title of the manuscript should be more specific. This article is of interest to the readers of this journal.

**We appreciate this statement of interest and suggestion. We agree and have modified the manuscript title to:**

**Modeling succinate dehydrogenase loss disorders in *C. elegans* through effects on hypoxia-inducible factor**

Additional editor questions:

1. Why was only the HIF-1alpha but not HIF-2alpha was proposed in the hypothesis? It is known that SDH loss is associated with HIF-2alpha changes.

**The editor is correct that humans have multiple HIFα subunits (HIF-1α, HIF-2 α, and HIF-3α) encoded by three separate genes, C. elegans is convenient in having only the single hif-1 gene. Thus, the genetics and physiology are simplified and the study refers only to hif-1 mutants, since this defines the complete HIF system in C. elegans. To clarify this important point, we have added the following sentence in the introduction, with a reference.**

**For example, while humans have three HIFα subunits (HIF-1α, HIF-2 α, and HIF-3α) encoded by three separate genes, *C. elegans* has only a single hif-1 α gene, facilitating conclusive genetic analysis [22].**

2. What is the mechanism how Punc-31 affects SDHB expression? The page number 6 is very hard to read and understand. The same to applies to Figure 5.

**We appreciate the opportunity to clarify the technical methods description on page 6. We have added the following introductory sentence so readers may better understand how the unc-31 promoter is used in the genetic constructs described in this manuscript. We now include the statement:**

**The *C. elegans* *Punc-31* promoter was used to drive expression of Hif transgenes or RNA interference constructs for SDH subunit knockdown, allowing these effects to be limited to neurons known to be important for egg-laying.**

3. Succinate measurement is not included, what is the reason for this?

**We appreciate the chance to answer this good question. Because loss of SDH genes is embryonic lethal in *C. elegans*, as in mammals, we used RNA interference to limit SDH gene knockdown to a small subset of neurons in which the unc-31 promoter is active. These cells represent a very small minority of cells of the intact hermaphrodite animal, and are not expected to contribute significantly to succinate levels in whole animals, even though those individual cells accumulate succinate that poisons egl-9 function. Thus, genetic rather than biochemical approaches are appropriate here. To clarify this point for readers, we now add the sentence on page 10:**

**We note that global succinate accumulation in whole worms is not expected for SDH knockdown under these conditions, as effects would be limited to the small subset of cells where *Punc-31* is active.**

4. HIF downstream genes should be shown in a way to see the effect of HIF-1alpha and HIF-2alpha.

**We appreciate this suggestion. As noted above, C. elegans has a single HIF-1α gene whose biology is well understood. Effects of hif-1 and egl-9 mutants on egg-laying and other hypoxic responses have previously been demonstrated. Furthermore, changes in HIF-responsive genes are only anticipated in the small number of unc-31 positive neurons so are not expected to cause a distinct change in global mRNA and protein levels.**

5. How is this work related to paraganglioma? While SDH is, this work may not be and therefore some information related to paraganglioma and therapies/incidence could be deleted. This information can be also reduced in the summary.

**We appreciate this suggestion. While the impetus for this work involves understanding approaches to modify the behavior of tumors such as paraganglioma, where SDH loss is a driver, we have now reduced the paraganglioma focus of the introduction by deleting various sentences with unnecessary details**.

6. References 6,7 and 9 should include original studies.

**We appreciate this suggestion. In the interests of the requested brevity and to ensure that the manuscript, as suggested, is not overly focused on PPGL, we have elected to retain the citation of these three review articles that we believe to nicely capture the relevant primary literature. We do include a number of essential primary references as well.**

7. von Hippel Lindau should be von Hippel-Lindau

**We appreciate this correction and have made the change**.

Reviewer: 1

It is clear from the paper that:

- SDHB KD is associated with egg retention
- This finding is linked to succinate through regulation of HIF1 stabilization
- Evaluation of girth provides an indirect reliable marker of succinate accumulation

In this way the *C. elegans* model could be a useful tool for screening drugs

**We thank the referee for this favorable review.**

1. A critical issue of this paper for translating results into PPGL would be that the link between PPGL tumorigenesis and HIF1 is still debated. Many experiments suggest that HIF2 is probably more important than HIF1. Is HIF2 also involved in egg-laying behavior? Please comment.

**We appreciate the opportunity to clarify. As noted in our response to editor summary question 1 above, the reviewer is correct that humans have multiple HIFα subunits (HIF-1α, HIF-2 α, and HIF-3α) encoded by three separate genes, and with ongoing research about which HIF protein(s) are relevant to PPGL biology. In contrast, *C. elegans* is convenient in having only the single hif-1 gene. Thus, the genetics and physiology are simplified and the study refers only to hif-1 mutants, since this defines the complete HIF system in *C. elegans*. This also means that the C. elegans model is a potential generic tool to study HIF effects without the need to differentiate between multiple HIF proteins. To clarify this important point, we note that we have added the following sentence in the introduction, with a reference.**

**For example, while humans have three HIFα subunits (HIF-1α, HIF-2 α, and HIF-3α) encoded by three separate genes, *C. elegans* has only a single hif-1 α gene, facilitating conclusive genetic analysis [22].**

2. Although intracellular succinate is very important in SDHB-related PPGL, it seems to be not only contained into the mitochondria and cytosol of the mutated cells but could act as an extracellular mediator, possibly via a specific receptor '"hormone like action". Did the authors assess the concentrations of intra-cellular and extra-cellular succinate (media) in SDHB KD models? Please add information. Would the results be similar if the media is removed very often during experiments? Please add information.

**We appreciate this insightful reviewer suggestion. As noted above in our response to the related editor summary question, because loss of SDH genes is embryonic lethal in *C. elegans*, as in mammals, we used RNA interference to limit SDH gene knockdown to a small subset of neurons in which the unc-31 promoter is active. These cells represent a very small minority of cells of the intact hermaphrodite animal, and are not expected to contribute significantly to succinate levels in whole animals, even though those individual cells accumulate succinate that poisons egl-9 function. Thus, genetic rather than biochemical approaches are appropriate here. To clarify this point for readers, we now add the sentence on page 10:**

**We note that global succinate accumulation in whole worms is not expected for SDH knockdown under these conditions, as effects would be limited to the small subset of cells where *Punc-31* is active.**

**The reviewer is correct that local “hormone-like” effects of succinate, limited to the immediate vicinity of the specific neurons where SDHB was knocked down, cannot be ruled out.**

3. Succinate accumulation may lead to acidific pH. Is pH controlled during experiments ? Is extra or intracellular pH influences egg laying behavior ? Please add information.

**We thank the referee for this insightful suggestion. We again note that that succinate accumulation is anticipated only within the small number of neurons where the unc-31 promoter is active, so there is not likely to be any substantial acidification at the organismal level. Moreover, the worms are cultured in NGM media containing phosphate buffer.**

Reviewer: 2

Comments:

This is an interesting manuscript documenting a link between SDH and biological activities of C. elegans. The link between one of the key enzymes of the Krebs cycle, SDH, that feeds electron into the OXPHOS system, and biology of a worm is intriguing.

**We thank the referee for this favorable review.**

1. Concerning the experimental set-up, the manuscript is well executed, although I have some concerns about presentation of results. Figure 4 is really difficult to read. Although there are significant differences, the individual data are too close to each other, so the authors may start the y-scale at the value of 0.5 or 0.75 rather than 0.

**We thank the reviewer for the endorsement of the experiments and the opportunity to improve the presentation. We have modified Fig. 4 as requested, departing from our usual convention that the y-axis must gave an origin of 0.0, and have adjusted the range to allow the data differences to be easier to appreciate.**

2. The Introduction needs to be re-written partially. It is a bit too long. Also, the authors should refer to a key recent review that documents the role of SDH (complex II, CII) in the Krebs cycle and the OXPHOS system, i.e. Bezawork-Geleta A et al. Mitochondrial complex II: At the crossroads. Trends Biochem Sci 2017, 42, 312-325.

**We appreciate this suggestion and have reduced the length of the introduction as noted in the response to the editor comments. We have also have now added citation to the very appropriate review suggested by the referee.**

3. The authors state in the Introduction that 'Loss-of-function mutation of any SDH subunit leads to a nonfunctional complex...' This is not exactly true. For example. loss of SDHB results in the assembly of the so called CII-low, a subcomplex of CII comprising SDHA and SDHAF2 and/orSDHAF4 subunits. This CII-low allows for slow rate tumor formation> This should be included in the manuscript, as well as the original reference, where this was shown, i.e. Bezawork-Geleta A et al. Alternative assembly of respiratory complex II connects energy stress to metabolic checkpoints. Nat Commun 2018, 9, 2221.

**We very much appreciate this comment by the referee and we now are pleased to correct our statement in the introduction and include citation to this important reference. The revised introduction statement now reads:**

**Loss-of-function mutations of SDH subunits lead to dysfunctional complexes {Bezawork-Geleta, 2017;Bezawork-Geleta, 2018}.**
